# A stakeholder workshop about modelled maps of key malaria indicator survey indicators in Madagascar

**DOI:** 10.1186/s12936-019-2729-7

**Published:** 2019-03-22

**Authors:** Rosalind E. Howes, Kaleem Hawa, Voahangy Fanomezana Andriamamonjy, Thierry Franchard, Raharizo Miarimbola, Sedera Aurélien Mioramalala, Jean Florent Rafamatanantsoa, Mirana Ando Mbolatiana Rahantamalala, Solo Harimalala Rajaobary, Hariniaina David Gaël Rajaonera, Andrianiaina Parfait Rakotonindrainy, Clairaut Rakotoson Andrianjatonavalona, Dina Ny Aina Liantsoa Randriamiarinjatovo, Faratiana Michèle Randrianasolo, Rado Malalatiana Ramasy Razafindratovo, Masiarivony Ravaoarimanga, Maurice Ye, Peter W. Gething, Cameron A. Taylor

**Affiliations:** 1 Malaria Atlas Project, Nuffield Department of Medicine, Big Data Institute, University of Oxford, Oxford, UK; 2 National Malaria Control Programme, Ministry of Health, Antananarivo, Madagascar; 3 Ministry of Health, Antananarivo, Madagascar; 4 Faculty of Science, University of Antananarivo, Antananarivo, Madagascar; 5 Department of Public Health, Faculty of Medicine, University of Antananarivo, Antananarivo, Madagascar; 6 Direction des Études et de la Planification, Ministry of Health, Antananarivo, Madagascar; 7 Direction du Système d’Information, Ministry of Health, Antananarivo, Madagascar; 8 Institut Pasteur de Madagascar, Antananarivo, Madagascar; 9 MEASURE-Evaluation, ICF, Antananarivo, Madagascar; 10 The DHS Program, ICF, Rockville, USA

**Keywords:** Madagascar, The DHS Program, Malaria Indicator Surveys, Malaria indicator maps, Geostatistical modelling, Workshop

## Abstract

The Demographic and Health Surveys (DHS) Program has supported three household Malaria Indicator Surveys (MIS) in Madagascar. The results of 13 key malaria indicators from these surveys have been mapped as continuous surfaces using model-based geostatistical methods. The opportunities and limitations of these mapped outputs were discussed during a workshop in Antananarivo, Madagascar in July 2018, attended by 15 representatives from various implementation, policy and research stakeholder institutions in Madagascar. Participants evaluated the findings from the maps, using these to develop figures and narratives to support their work in the control of malaria in Madagascar.

## Background

The Demographic and Health Surveys (DHS) Program in Rockville, Maryland USA, funded by USAID, has provided technical assistance to more than 300 household surveys in over 90 countries, advancing global knowledge of health and population trends in developing countries [1]. The most recent nationally-representative surveys conducted in Madagascar are the 2011, 2013 and 2016 Malaria Indicator Surveys (MIS) [2–4]. Following the 2016 MIS, there was a request to further examine the outputs from these surveys and aid in the capacity strengthening of the participants to use the data for programmatic decision-making. Given the diverse epidemiology of malaria in Madagascar and issues of accessibility across the country [5–7], it was proposed to collaborate with the Malaria Atlas Project (MAP) to create a series of modelled surface maps showcasing the results of 13 key malaria indicators from both the 2013 and 2016 MIS surveys [8]. The MAP research group is a World Health Organization (WHO) Collaborating Centre in Geospatial Disease Modelling that uses an evidence-based cartographic approach to model continuous spatial maps and quantify metrics including population estimates of malaria infection prevalence and clinical burden [9–11]. At the time of writing, modelled indicator maps for 32 different country surveys globally are freely available from the DHS Spatial Data Repository website.

The spatially modelled surfaces of the DHS malariometric data are produced using a combination of publicly available DHS data and global environmental datasets, and use standardized geostatistical methods to promote comparability across countries and facilitate policy and programme decision-making. Although the creation of these surfaces is not new, their incorporation as part of formal decision-making processes is not yet routine.

To help contribute to informed decision-making about future malaria policies and intervention programmes in Madagascar, The DHS Program and MAP collaborated to create a workshop curriculum exploring the spatially modelled surfaces of the 2013 and 2016 MIS surveys. The workshop goals were to convey the added value of spatial modelling in deriving more robust indicator metrics, and to help support the integration of maps into monitoring and evaluation of malaria indicators across Madagascar.

## Why map the MIS indicators?

Improved understanding of geographic variation and inequity in health status, wealth, and access to resources within countries is increasingly recognized as central to meeting malaria control targets [12]. Malaria indicators assessed at national levels may conceal important inequities in smaller administrative/geographic areas, often with the rural poor the least well represented. As malaria prevalence drops and international funding for malaria comes under pressure, the ability to target limited resources to underserved groups becomes more crucial [13, 14]. At the same time, gaps exist in progress towards achieving targets for key malaria indicators [15]. Monitoring inequalities for targeting control interventions and measuring progress towards health and development goals requires a reliable, detailed, and disaggregated evidence base.

Different approaches currently allow for estimating malaria indicator metrics for small geographic units. These include (i) increasing the sample sizes of national household surveys to ensure representative sampling of lower administrative areas, (ii) using data from routine health information systems from health facilities or communities, and (iii) small area estimation including spatially interpolated maps that use statistical modelling techniques to predict values for small geographic units. Increasing sample sizes incurs additional costs and time, which are often not feasible in an increasingly resource-constrained environment. Next, the quality and national representativeness of routine health information system data is not always reliable, nor are the data always easily accessible. It is, therefore, the third approach with spatial interpolation that has attracted increased interest in recent years [12, 16, 17].

Malaria is highly diverse across Madagascar, requiring different combinations of interventions across the country’s epidemiological ecozones [5, 18]. These areas are stratified based on the duration and intensity of transmission, and the preceding years’ diagnostic positivity rates. The Madagascar MIS sampling design was powered to generate summary indicator metrics at these epidemiological and programmatic scales (n = 5), as well as at the national level. Programme implementation, however, is managed at the health district level (n = 114), and the raw MIS results cannot determine indicator progress at this scale. Geostatistical approaches are able to use the available cluster-level MIS results coupled with environmental covariates to predict indicator values for all areas of the country. This greatly empowered dataset allows indicators to be aggregated to programmatically useful scales [8].

## Workshop structure and objectives

The purpose of the Madagascar Modelled Surfaces Workshop was to assist the National Malaria Control Programme (NMCP, or “Direction de Lutte contre le Paludisme”) and other malaria data partners in the interpretation and application of modelled maps that showcase the geographic variation of malaria indicators across Madagascar. The workshop’s specific objectives included training on (i) understanding and correctly interpreting the core household MIS indicators, (ii) understanding the creation of the modelled surfaces, their limitations and inherent assumptions, (iii) accurately interpreting the modelled surfaces, and (iv) identifying narratives in the maps to answer key programmatic questions.

In advance of the workshop, key members of the NMCP, the Ministry of Health (MoH), the Direction des Etudes et de la Planification, the Direction du Système d’Information, and the research organization Institut Pasteur de Madagascar were contacted to nominate members of their staff to attend the workshop. Participants were required to have some experience with geographic information system (GIS) software and needed to analyse malaria data as part of their job. In total, 15 people participated in the workshop, which took place over 4 days in July 2018 in Antananarivo, Madagascar.

Activities throughout the workshop were designed to encompass a range of adult learning techniques. Interactive PowerPoints, blended learning, guided demonstrations, hands-on exercises and small group activities were all used. The workshop culminated in final presentations from each indicator topic team (broadly categorized as: vector, case management, and morbidity) including a programmatic question they wanted to address, the audience for their presentation, an introduction to the problem in Madagascar, the indicators selected for analysis, appropriate maps, and interpretation/recommendations emerging from the modelled surfaces.

## Mapping methodology

The field of spatial statistics is continually developing ever more complex and refined models [12, 16]. Highly bespoke approaches, however, limit the comparability of model outputs, both through time and between locations. Instead, the methodological approach for mapping The DHS Program indicators was deliberately designed to generate standardized outputs informed by globally available input datasets, thereby allowing full comparability across countries and survey years [8]. From the Madagascar MIS results, a subset of 13 malaria indicators was identified as suitable for spatial analysis [19], and were modelled in advance of the workshop (Table 1). These surfaces (including both mean predictions and the associated 95% credible interval uncertainty maps) are all freely available from The DHS Spatial Repository website, together with detailed reports about the mapping methods employed [17, 19–22]: http://spatialdata.dhsprogram.com/modeled-surfaces/. Globally, modelled maps of selected indicators from 32 MIS/DHS surveys from 31 countries are currently available.

**Table 1:** MIS indicators from the 2013 and 2016 Madagascar surveys selected for spatial modelling

Indicator	Definition
MLFEVTCACT	Among children under age five with fever in the 2 weeks preceding the survey, the percentage who took a drug combination with artemisinin
MLFEVTCADV	Among children under age five with fever in the 2 weeks preceding the survey, the percentage for whom advice or treatment was sought
MLFEVTCBLD	Among children under age five with fever in the 2 weeks preceding the survey, the percentage who had blood taken from a finger or heel for testing
MLHEMOCHL8	Percentage of children aged 6–59 months with haemoglobin lower than 8.0 g/dl
MLIPTPW2SA	Percentage of women aged 15–49 with a live birth in the 2 years preceding the survey who during the pregnancy took two or more doses of SP/Fansidar, with at least one dose during an antenatal care visit [intermittent preventive treatment for pregnant women (IPTp2+)]
MLIRSMHIRS	Percentage of households with indoor residual spraying (IRS) in the 12 months preceding the survey
MLITNAPACC	Percentage of the de facto household population who could sleep under an ITN if each ITN in the household were used by up to two people
MLNETCCITN	Percentage of children under age five who slept under an ITN the night before the survey
MLNETPHITN	Percentage of households with at least one ITN
MLNETUPITN	Percentage of the de facto household population who slept under an insecticide treated net the night before the survey
MLNETWWITN	Percentage of pregnant women who slept under an ITN the night before the survey
MLPMALCMSY	Percentage of children aged 6–59 months tested using microscopy who are positive for malaria
MLPMALCRDT	Percentage of children aged 6–59 months tested using a rapid diagnostic test (RDT) who are positive for malaria

The modelled continuous surfaces together with 95% credible interval maps are available for both survey years as .png images and .tif raster files from the DHS Program Spatial Data Repository [21, 22]

As previously described, a model-based geostatistics (MBG) approach was found most appropriate for generating standardized spatial outputs from the raw MIS cluster results [8, 20]. The foundation of MBG is spatial interpolation, where estimates for each grid cell are driven by nearby survey observations coupled with the geographic patterns of biologically pertinent spatially-continuous covariate surfaces (Table 2). The modelling process characterizes the patterns observed in the survey data into four components: sampling variance is represented by a binomial sampling model; non-sampling error is explained through fixed effects (by a multivariate regression relationship defined by linking the indicator variables to the covariates) and random effects (Gaussian Process parameterized by a Matern spatial covariance function); and finally, a simple Gaussian noise term represents residual variation. All model parameters are jointly estimated in a Bayesian framework [8, 20] which generates pixel-level predictions for each indicator across the country, informed by the patterns in the relevant environmental covariates selected by the model for their spatial correlation with the raw indicator data.

**Table 2:** Mapping covariates used in the modelling [17]

Short name	Description	Original data source	Temporal	Date
Population				
Access	Travel time to cities with greater than 50,000 population via all transport methods	http://forobs.jrc.ec.europa.eu	Static	2000
NTL	VIIRS nighttime lights	http://ngdc.noaa.gov/eog/	Static	2012
GPW	Gridded population of the world (GPW) population density	http://sedac.ciesin.columbia.edu/	Static	2010
Physical earth				
Elevation	Shuttle radar topography mission (SRTM) near—global digital elevation models (DEMs)	http://webmap.ornl.gov/	Static	2000
Environment				
Aridity	Mean annual aridity	http://csi.cgiar.org/Aridity/	Synoptic	1950–2000
PRECIP	Average monthly rainfall	http://www.worldclim.org/	Synoptic	1950–2000
EVI	Enhanced vegetation index	http://modis.gsfc.nasa.gov/	Multitemporal	2001–2014
LST.day	Land surface temperature in the daytime	http://modis.gsfc.nasa.gov/	Multitemporal	2001–2014
LST.delta	Land surface temperature daily fluctuation range	http://modis.gsfc.nasa.gov/	Multitemporal	2001–2014
LST.night	Land surface temperature in the nighttime	http://modis.gsfc.nasa.gov/	Multitemporal	2001–2014
PET	Mean annual potential evapotranspiration	http://csi.cgiar.org/Aridity/	Synoptic	1950–2000
TCB	Tasseled—cap brightness	http://modis.gsfc.nasa.gov/	Multitemporal	2001–2014
TCW	Tasseled—cap wetness	http://modis.gsfc.nasa.gov/	Multitemporal	2001–2014

These overarching methodological concepts were introduced during the workshop, together with a discussion of exploratory spatial data descriptive statistics (including variograms and histograms plotting the structure of the indicator values) and model validation statistics. The workshop objective was to provide conceptual insight into the mapping methods and allow appropriate critical evaluation of the modelled outputs. As such, strong emphasis was also placed on the output limitations (e.g. weakness of modelling urban areas and difficulties of temporality in the indicator measures [17]) and the importance of assessing relative confidence in the predictions between areas. Examples of GIS-based manipulations of the modelled surfaces were then presented and trialed by the participants.

## Putting maps into practice: exploratory case studies of programmatic applications of the modelled MIS indicator maps

The final two days of the workshop were largely dedicated to allowing the participants to explore the modelled maps to derive programmatically-pertinent recommendations that could plausibly be applied in the context of their current positions. Outcomes of these group discussions are summarized here, illustrating different ways in which The DHS Program modelled surfaces may be easily and rapidly applied by NMCPs and fellow stakeholders. While more formal analyses were encouraged, these were outside the timeframe of the workshop.

### *Example 1*

Strengthening access to intermittent preventative treatment in pregnancy.

**Fig. 1:**
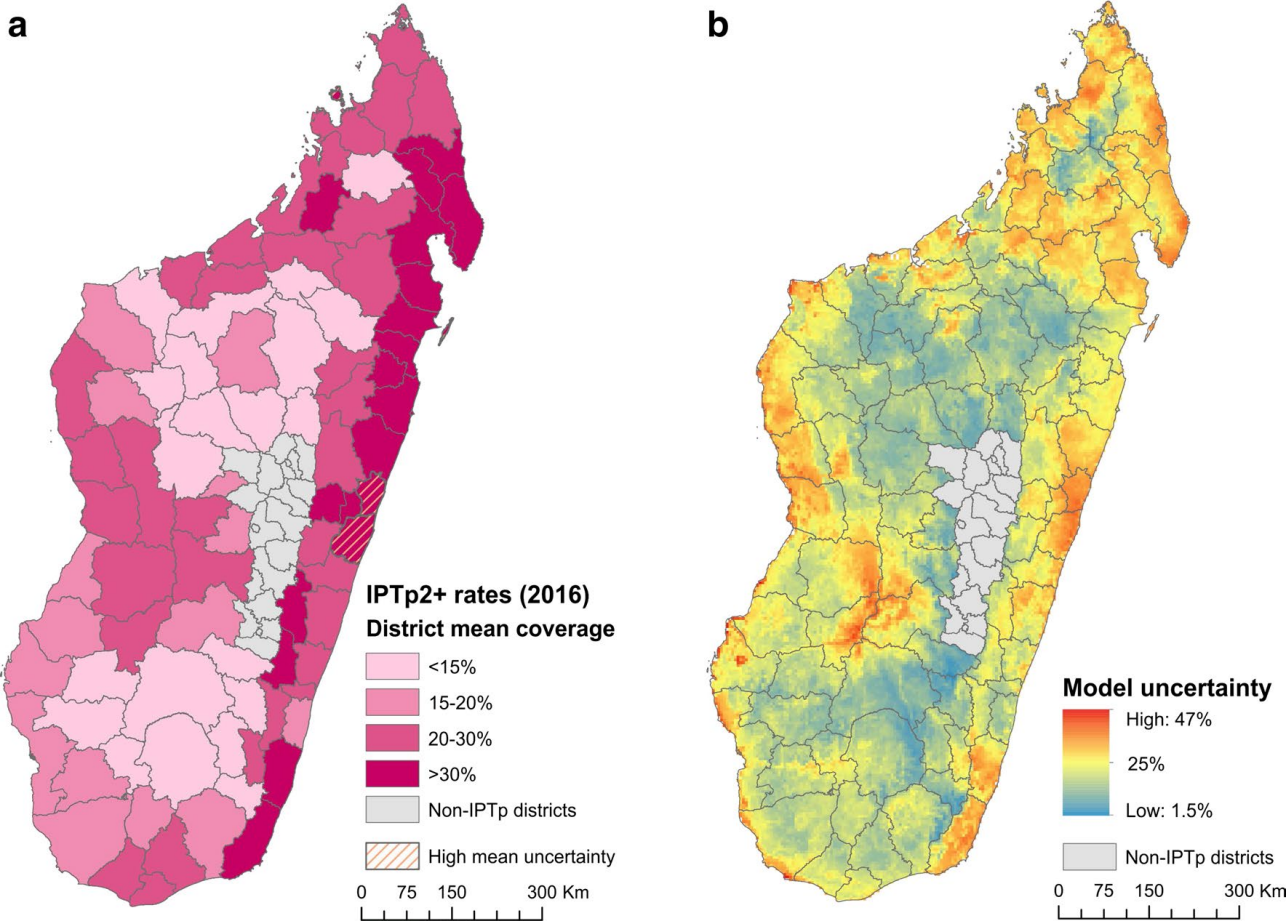
Coverage in 2016 of women with a live birth in the 2 years preceding the survey who received two or more prophylactic doses of SP/Fansidar (IPTp2+). The spatially continuous map is summarized to the district level (mean values of indicator ML_IPTP_W_2SA) in **a**, with pixel-level uncertainty shown in **b**

Intermittent preventative treatment in pregnancy (IPTp) with sulfadoxine–pyrimethamine (SP) was brought into policy in Madagascar in 2004, and a progressive scale-up has led to this now being implemented in all control-phase districts. The WHO recommendation to increase the minimum number of doses from two (IPTp2+) to three (IPTp3+) was initiated in Madagascar in 2015. Given this, the IPTp3+ coverage indicator was not appropriate to examine given the temporality of its definition (having as denominator the “total number of women surveyed who delivered a live baby in the 2 years preceding the survey” [23], i.e. extending to before the IPTp3+ policy was locally implemented). Instead, IPTp2+ was evaluated. District-level aggregation of the modelled surface indicated that coverage in 2016 remained below 20% in 40% of target districts (Fig. 1a). This was consistent with relatively weak coverage of antenatal consultations with an estimated one third of pregnant women never attending an antenatal consultation (source: MoH, 2017), and health facilities regularly notifying SP stock-outs (43% did during 2016; source: NMCP, 2017).

The modelled surfaces made apparent a degree of spatial heterogeneity in the coverage of IPTp2+ (Fig. 1a), with high predictive uncertainty (> 20%) also widespread (Fig. 1b) likely associated with the relatively small sample sizes inherent to this indicator (N = 2786, relative to 10,816 respondents for other indicators in 2016).

Recommendations were made for increased sampling effort in high uncertainty coastal districts during future MIS, as well as strengthening the reporting of IPTp during antenatal consultations at health facilities. Reinforced collaboration between the NMCP and the National Family Health programme was also recommended, alongside renewed awareness campaigns targeted to areas of weakest coverage (Fig. 1a).

### *Example 2*

Spatio-temporal trends in access to insecticide-treated nets and implications for future mass distribution campaigns.

Madagascar aims for universal coverage of insecticide-treated nets (ITN) across all control-phase districts. This is achieved primarily through mass distribution campaigns, the last having been in 2012–2013, 2015 and 2018 [18, 24]. The current objective is that at least 90% of households in the target districts should have at least one ITN per two residents. Several channels of continuous distribution supplement the mass campaigns, including antenatal consultations, community-health worker distributions, and subsidized sales in peri-urban communities. The MIS in 2013 and 2016 therefore assessed the overall impact of these activities, allowing changes in coverage during that time period to be quantified.

Interpretation of the survey outcomes must account for timings relative to mass distribution campaigns. The 2013 MIS took place part way through a distribution campaign, with 31 districts covered in the 6 months before the MIS, and 61 districts after the MIS. In contrast, all target districts were included in the mass ITN distribution during the 6 months preceding the 2016 MIS.

**Fig. 2:**
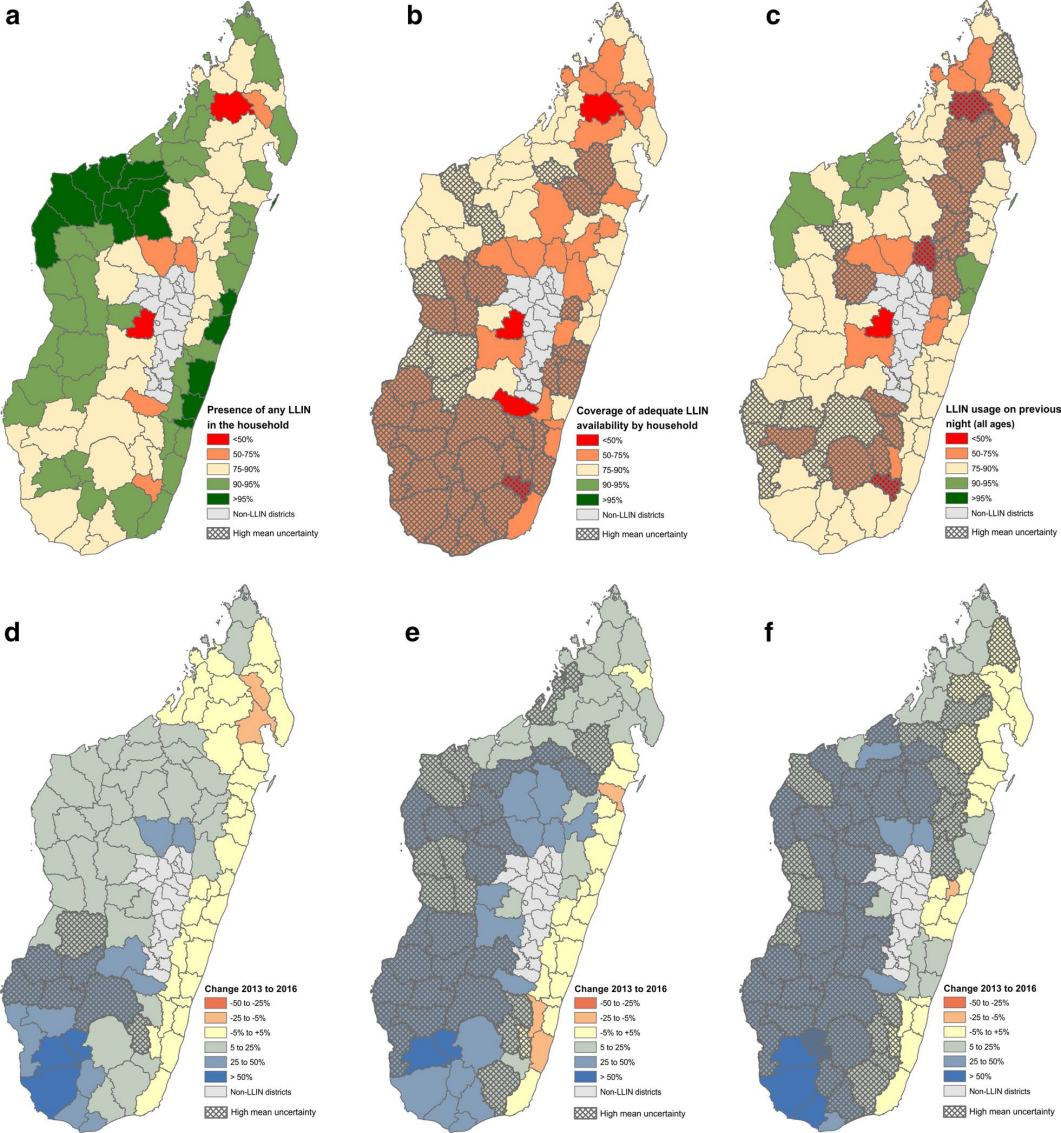
Characteristics of ITN coverage and usage at the district level for 2016 (**a**–**c**) and relative change from 2013 (**d**–**f**). **a**, **d** Correspond to the percentage of households with at least one ITN (indicator ML_NETP_H_ITN). **b**, **e** Quantify the proportion of the population with access to an ITN within their household when shared by at most two people (ML_ITNA_P_ACC). **c**, **f** Map the percentage of household residents reported to have slept under an ITN the night preceding the interview (ML_NETU_P_ITN). The spatially continuous maps are aggregated to the district level and presented as mean values, with relative uncertainty based on crude means of the pixel-level uncertainty metrics

Several ITN coverage indicators based on different denominators (households vs. residents) were included in the spatial analyses, each representing different aspects of the programme’s impact (Table 1). Here, participants selected three of these indicators to assess ITN coverage across Madagascar in 2016 (Fig. 2a–c) and relative changes in these indicator levels since 2013 (Fig. 2d–f), with the aim of exploring what lessons could be derived from the modelled surfaces for future distribution campaigns. These included the spatial reach of the distribution campaigns using the indicator of presence of any ITNs in the household (Fig. 2a, d), the adequacy of coverage when accounting for the number of household residents (the target is for one net per two individuals; Fig. 2b, e), and finally usage of available nets (Fig. 2c, f). The mapped surfaces were aggregated to district units to reflect the level at which decision-making and logistics are coordinated during ITN campaigns.

The modelled surfaces revealed that while the reach of the ITNs was quite high, with 52 of 92 target districts predicted to have > 90% of households with at least one bed net, this coverage dropped dramatically when considering the adequacy of ITN availability according to the objective of one ITN per two people by household. No district met the national objective of 90% in 2016, though 39 (42%) had levels > 75%. Nevertheless, reported indicators of usage by household residents indicated that ITNs were being used at rates suggesting that even where ITN numbers were insufficient within households, residents were sleeping under the nets which were available. Coverage of these ITN indicators was generally better in coastal areas where transmission was higher [6], particularly along the northern districts of the west and east coasts. Coverage dropped into the highland areas where transmission was less intense. The maps suggested generally sustained or positive changes in coverage between 2013 and 2016, with greatest improvements in southern districts even though coverage remained among the lowest nationally in these same districts. In the east coast districts, where transmission is highest and ITN coverage still falls short of national targets, there was little reported change from 2013. However, there were extensive areas of high uncertainty in the model predictions, notably in the maps of ITN accessibility and usage where the map estimates need to be interpreted with caution (Fig. 2b, c, e, f). Spatial heterogeneity in the cluster-level results may explain uncertainty in these areas.

Recommendations for future campaigns that emerged from these modelled maps were to focus on increasing the numbers of nets distributed, with reinforced efforts particularly in east coast areas where coverage was low despite relatively high transmission. Results on usage were encouraging but still inadequate, indicating that further behaviour communication interventions would be important alongside the distributions, as per the NMCP’s guidelines.

### *Example 3*

Treatment-seeking for febrile infants.

Seeking treatment during a febrile episode is the critical first step towards effective case management and reductions in malaria morbidity, as well as towards ensuring reliable reporting of malaria episodes for surveillance. Low treatment-seeking rates across much of Africa are a main reason for the WHO using data sources independent of routine surveillance in their estimations of clinical case burdens [15, 25]. The MIS indicator quantifying this is rates of treatment seeking by mothers for any children younger than 5 years having suffered a febrile episode in the 2 weeks preceding the survey.

The national-level MIS results from Madagascar suggest an increased, but nevertheless low, treatment-seeking rate from any type of health provider for febrile children from 38% in 2013 to 46% in 2016. Only 29% and 36%, respectively, sought treatment from the public health facilities likely to provide appropriate free case management and to report monthly case estimates to the centralized MoH database. These low rates of contact with recommended healthcare providers are a target of Madagascar’s current National Strategic Plan through behaviour-change communication activities. Better insight into this indicator’s tendencies would help focus future efforts based on current gaps and local infection risk levels.

**Fig. 3:**
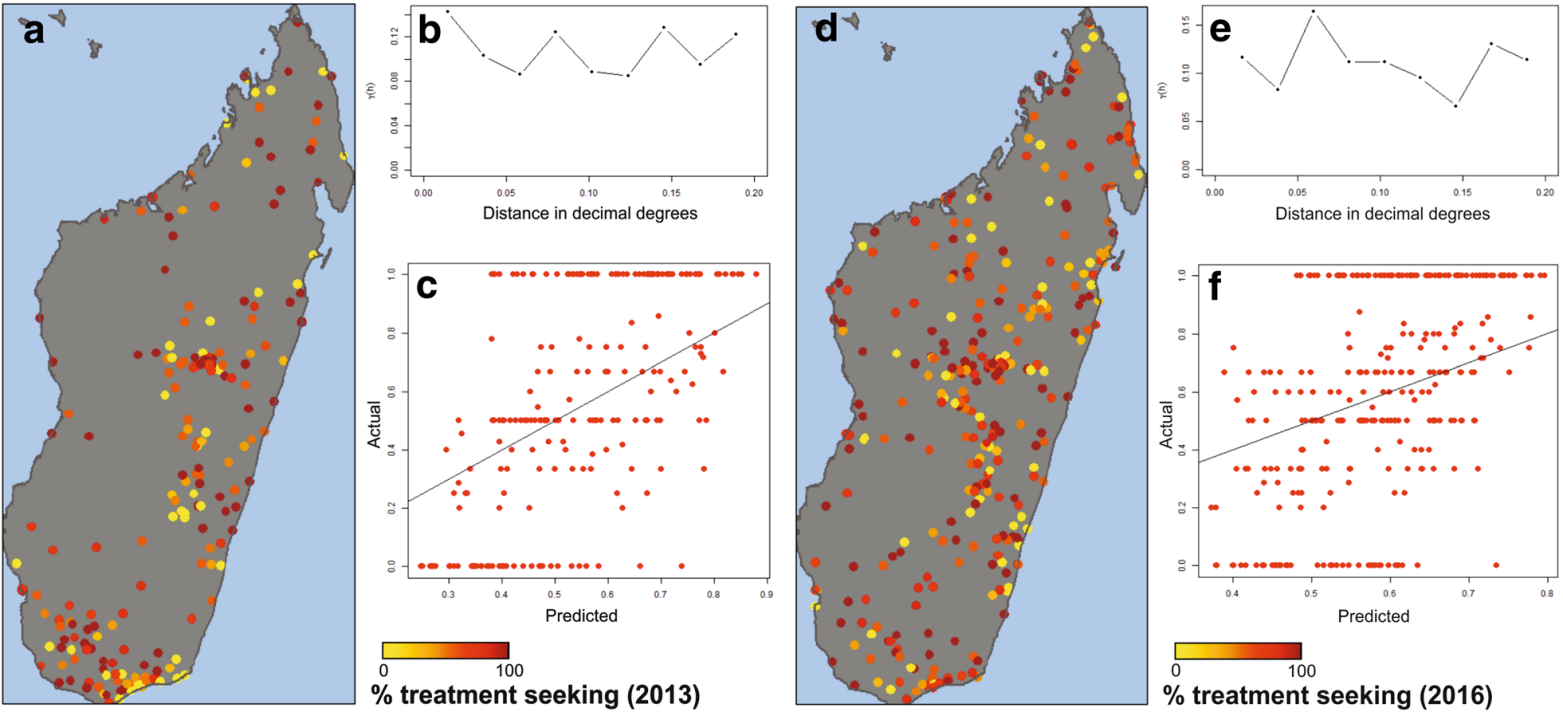
Descriptive statistics of the spatial characteristics of treatment seeking rates by mothers for febrile infants under 5 years old in the 2 weeks preceding the interview (indicator ML_FEVT_C_ADV). **a**–**c** Represent the 2013 MIS data, and **d**–**f** are for 2016. The maps in **a**, **d** illustrate the cluster-level raw treatment-seeking rates, while **b**, **e** are variograms (a tight spatial structure would show an increasing lag—or dissimilarity between points—with increasing spatial distance). **c**, **f** Plot the model validation of observed cluster-level raw values (y-axis) against predicted values in those locations. In the model validation, a random 25% of the dataset is withheld and the model run with the remaining 75%; this process is repeated four times without replacement, thus giving validation-predictions for all cluster locations (**c**, **f**). Tight scatterplot correlation suggests greater precision in the model’s predictive performance

The MIS treatment-seeking indicator was therefore evaluated to see what spatio-temporal trends could be derived beyond the national summary figures. Descriptive statistics are presented for 2013 (Fig. 3a–c) and 2016 (Fig. 3d–f). The cluster-level raw numbers showed a high level of spatial heterogeneity (Fig. 3a, d), likely associated with the variable and sometimes small samples sizes (overall n_2013_ = 633 and n_2016_ = 1096 eligible mothers across the country whose children had suffered a febrile episode in the 2 weeks preceding the survey, which become very small when considered at the cluster-level). The raw datapoint values (Fig. 3a, d) and the variograms (Fig. 3b, e) revealed that the dataset had limited spatial structure, also reflected by high relative uncertainty in the predicted maps. Consistent with these characteristics, the model predictions revealed low correlation with the raw cluster-level observed data (Fig. 3c, f). These warnings in the data suggest that the spatial predictions from this current dataset and model may not be appropriate to rely on.

These insights indicate to stakeholders interested in improving rates of appropriate malaria case management that the data and models examined here are insufficient to allow meaningful assessments of current treatment-seeking levels. Additional efforts will be necessary to strengthen the evidence base and allow this indicator’s subnational trends to be understood. The low sample sizes associated with this specific MIS indicator—owing to its opportunistic nature—limit the statistical power required for high-resolution analyses.

Alternative approaches, such as active case detection, or simply larger sample sizes could provide more robust insight into this important indicator. A strong message, therefore, emerges to advocate for reinforcement of this indicator, to allow important questions about variability in behaviour across the island and the impact of NMCP initiatives on behaviour change over time to be answered. This remains essential to improving treatment-seeking rates and appropriate case management: cornerstones of any control programme.

## Participant evaluations

Qualitative interviews conducted ahead of the workshop with NMCP staff suggested that knowledge and use of maps by the Programme were commonly limited to descriptive mapping of aggregated reported incidence rates and summaries of epidemic clusters. Modelled malaria risk maps were not used programmatically, nor was there significant knowledge of what they represent.

Participants were issued a 13-question test to evaluate their knowledge of MIS indicators, modelled surfaces, and statistical uncertainty both before and after the workshop. The mean participant score increased from 54 to 87%, with all but one participant improving in their performance (that participant’s score remained constant at 86%). Anonymous evaluation forms were also filled out on the last day of the workshop, giving participants the opportunity to assess the relevance, pace and content of the workshop content, and to give comments or suggestions. Feedback was positive. Participants indicated that they learned a great deal from the lectures and exercises. They appreciated the knowledge and skills acquired during the workshop and planned to use the modelled surfaces as part of programmatic decision-making in the future.

Throughout the workshop, participants identified examples of how the modelled maps could be applied in their specific domains of work. These included, for example, allowing more precise decision-making in the absence of complete datasets; tailoring intervention responses appropriately during epidemics using regionally-specific indicator information; providing more information in resource-constrained contexts where additional data is difficult to request; and use as advocacy tools when communicating with non-expert audiences or for synthesizing information in grant applications. The imminent launch of the 2018 bed net distribution campaign provided a very explicit case study for how the modelled surface maps could support refinements to future planning activities, with the maps highlighting areas at greatest need for reinforced resources.

The most consistent critique was that the workshop was not long enough. In particular, the majority of participants thought that they would have benefitted from more time to practice manipulating the maps in ArcGIS, as well as training in R coding. The workshop allowed participants the opportunity to discuss data-informed decision making in Madagascar, and many commented that the workshop helped further their understanding of malaria indicators which they deemed essential for programme level decisions. Discussions around the context, strengths and weaknesses of the indicators, as well as MIS study designs, proved to be a very valuable asset to the workshop.

## Conclusions

This was the first country-specific Modelled Surfaces Workshop to be implemented by The DHS Program. While this type of workshop may not be recommended for every country, it was highly beneficial for Madagascar, where multiple MIS surveys have been implemented and the malaria epidemiology is variable across the country. Developing the portfolio of modelled indicator maps and training the workshop participants to critically evaluate and analyse them, increases the capacity of the country’s malaria control stakeholders to make data-driven decisions. The Madagascar maps are freely accessible from the DHS Spatial Data Repository website [21, 22], alongside maps of selected indicators from MIS/DHS surveys in 30 other countries. All participants recommended the workshop to other NMCPs, and some requested additional training to be conducted in Madagascar.

## Abbreviations

DHS: Demographic and Health Surveys
GIS: geographic information system
IPTp: intermittent preventative treatment in pregnancy
ITN: insecticide-treated net
MAP: Malaria Atlas Project
MBG: model-based geostatistics
MIS: Malaria Indicator Survey
MoH: Ministry of Health
NMCP: National Malaria Control Programme
SP: sulfadoxine–pyrimethamine
WHO: World Health Organization
